# Environmental Efficiency Evaluation of China’s Power Industry Based on the Two-Stage Network Slack-Based Measure Model

**DOI:** 10.3390/ijerph182312650

**Published:** 2021-11-30

**Authors:** Wei Wei, Shuangying Ding, Silin Zheng, Jingjing Ma, Tong Niu, Jinkai Li

**Affiliations:** 1 Center for Energy Environment & Economy Research, School of Tourism Management, Zhengzhou University, Zhengzhou 450001, China; weiwei123@zzu.edu.cn (W.W.); dsy929890127@gs.zzu.edu.cn (S.D.); z18208131474@163.com (S.Z.); majingj926@126.com (J.M.); 2 Yellow River Institute for Ecological Protection & Regionally Coordinated Development, Zhengzhou University, Zhengzhou 450001, China

**Keywords:** power industry, environmental efficiency, NSBM model, power system reform

## Abstract

How to achieve the continuous improvement of the environmental performance level of the power industry within the requirements of clean and low-carbon energy development is the fundamental requirement and inevitable choice for the construction of ecological civilization and sustainable development. From the perspective of environmental protection, based on the Data Envelopment Analysis (DEA) method and the internal mechanism of power system production and supply, the power industry environmental efficiency evaluation index system was constructed, and the two-stage Network Slack-based Measure (NSBM) model considering undesired output was used to calculate China’s 30 provinces and municipalities from 1998 to 2019. The environmental efficiency is divided into two links: power generation efficiency and transmission and distribution efficiency. The study found that, within the research interval, the overall environmental efficiency of China’s 30 provinces is low, and the differences between provinces and cities are large, but they have gradually developed in a better direction after 2015. The power generation efficiency of the first link in most provinces and municipalities is higher than the transmission and distribution efficiency of the second link, and the low transmission and distribution efficiency is an important reason for the low comprehensive level of environmental efficiency. The overall evolution trend of environmental efficiency in the six regions of China is roughly the same, but the regional differences are obvious, showing a trend of “high in the southeast and low in the northwest”. The economic and natural resource differences in different provinces and cities in each region have led to varying degrees of redundancy in five aspects, including investment in power assets, installed power generation capacity, and length of transmission lines, which seriously affect the environmental efficiency of the power industry. This research attempts to open the “black box” of the environmental efficiency conversion process of the power industry, which can provide directions and strategic suggestions for the improvement of the efficiency of the power industry in China.

## 1. Introduction

The power industry is one of the most important energy-based industries for national development. It is the foundation and guarantee of modern social progress and national energy security. It is also one of the main reasons of environmental pollution caused by the consumption of scarce fossil energy, such as coal. Affected by COVID-19, China’s entire society’s electricity consumption in 2020 was 7.511 trillion kilowatt-hours, and the growth rate of electricity demand has declined, with a year-on-year increase of 3.1%, but it is still on the rise compared with 2019. The installed capacity of power sources nationwide was 2.201 billion kilowatts, a year-on-year increase of 9.5%, of which coal-fired power generation capacity accounted for 56.58%, and clean energy accounted for 43.42%. Among them, coal-fired power generation emits a large amount of pollutants, such as carbon dioxide, sulfur dioxide, nitrogen oxides, soot and dust, and mercury gas, which brings tremendous pressure to carbon emission reduction, mercury emission, public health, environmental governance [1], and the realization of ecological civilization in China’s power industry.

To this end, China has committed and formulated the goal of peaking carbon dioxide emissions around 2030 and achieving carbon neutrality by 2060 and plans to increase the proportion of non-fossil energy in primary energy consumption to about 20% by 2030 and decrease the ratio of carbon dioxide emissions per unit of GDP by 60~65% in 2005. At the same time, China will strictly control coal power projects, strictly control the growth of coal consumption during the “14th Five-Year Plan” period, and gradually reduce it during the “15th Five-Year Plan” period [2]. The main tasks of the new round of power system reform in 2015 and the “14th Five-Year Plan” are to grasp “greenness”, improve efficiency, point out that clean and low-carbon energy is the future direction of energy development, and strive to reduce environmental pollution and improve public health [3]. Environmental efficiency has been a research hotspot in academic circles in recent years, and it is also an important starting point for further achieving environmental friendliness and building an ecologically civilized city [4,5]. In view of the current environmental efficiency of China’s electric power industry, which is mostly at a low level, there are also problems between the various departments of electric power management, which is not coordinated, and internal responsibilities, which are not clear; it is particularly important to study the environmental efficiency of the industry. Therefore, this article will study how to achieve a balanced and sustainable development of China’s power industry while improving efficiency and protecting the environment. It is not only conducive to in-depth understanding of the power industry’s operating mechanism and technical efficiency change characteristics in China’s power system reform but also has practical guiding significance and practical value for promoting sustainable development in China’s new round of power market reform.

## 2. Literature Review

Since the implementation of the power industry regulatory reform in the 1960s and 1970s and the marketization reform of the power industry in China in 2002, the efficiency of the power industry has gradually been extensively studied. The commonly used method is data envelopment analysis (DEA) to measure the efficiency of the power industry [6,7]. Scholars in the field have done less research on specific issues in China. In addition to the increasingly serious power shortage and the advancement of power system reform in China in the early days, scholars have begun to pay close attention to the efficiency of China’s power industry, and the research mainly focuses on the efficiency and influencing factors of power generation, transmission and distribution, or the overall power industry. 

In relation to the researches of technical efficiency in the power industry, as early as 2009, Maruyama et al. studied the time series data of power efficiency in 138 countries and regions from 1971 to 2005. The results show that the overall efficiency improvement is gradual, and there are significant differences between regions and countries [8]. Barbose et al. (2014) studied an innovative project in the western United States that aims to meet power demand and reduce carbon dioxide emissions and incorporate energy efficiency into regional transmission planning activities to achieve effective integration of power efficiency [9]. Studies have shown that excessive use of fuel will reduce the efficiency level of the power generation industry, and considering the importance of resources and the environment to the power industry, some scholars have broken through the traditional efficiency evaluation of the limitation of emphasizing economic benefits over sustainable development, incorporating carbon dioxide emissions as an input indicator into the DEA model [10]. Since then, scholars have continuously added carbon dioxide, sulfur dioxide, soot, and nitrogen oxides as undesired outputs into the DEA model [11] to comprehensively analyze the environmental efficiency of the power industry [4,12,13,14,15,16,17]. Chen et al. (2017) evaluated the energy efficiency of China’s inter-provincial power industry under environmental constraints and found that the energy efficiency of each province did not increase significantly from 2005 to 2014, and it has a certain degree of volatility [4]. Du et al. (2015) estimated the environmental efficiency, emission reduction potential, and edited emission reduction cost of carbon dioxide emissions from coal-fired power plants in China, which showed that the proportion of coal consumption has a negative impact on the environmental efficiency of power plants [14]. Some scholars have also paid attention to mercury emission from industrial activities as a great threat to public health and ecosystems [1]. As regards developing new strategies and materials to remove mercury, the development of efficient and low-cost mercury adsorbents still remains a serious task. Meanwhile, it is also an important demand in the field of energy and environment. Jia et al. (2021) found that doped metal oxides can play a synergistic role in enhancing the mercury removal performance of the modified biochar [18], and this shows that the development and application of related technologies and methods can help to improve efficiency. Song et al. (2020) used the super-efficiency DEA method to measure China’s power efficiency level from 2009 to 2017 and explored the trend of efficiency changes before and after China’s supply-side reforms, indicating that the efficiency after reforms showed a fluctuating upward trend, including technological progress, economic development, and opening to the outside world, positively affecting power efficiency [19]. Although the above studies incorporate various pollution factors that affect environmental quality into the model, only one or two of them are considered, which lacks comprehensiveness. As for the power transmission and distribution industry, there are few studies on its efficiency. The main research objects are provincial power companies, power supply companies, and the State Grid Corporation of China [20,21,22]. Li et al. (2014) used DEA and super-efficiency DEA models to measure the economic efficiency and unified efficiency of 24 power supply companies in China on the basis of considering expected and undesired output. The results show that unified efficiency is used to evaluate power supply companies. It will encourage power supply companies to pay more attention to social and environmental benefits while paying attention to economic benefits [22]. There are also scholars studying the investment efficiency of inter-provincial grid companies based on different perspectives [23].

Most of the above studies adopt the traditional DEA model, which can only analyze the overall efficiency of the decision-making unit and regard the production process as a “black box”. The inability to deeply study the influence of the intermediate production process or the internal structure of the decision-making unit on the overall efficiency will lead to inaccurate efficiency evaluation. The power system is a complex, organic whole composed of multiple departments. The input-output variables of each department are not simply involved in the production activities of one department. If the traditional black box model is adopted, it can only analyze the production status of the power industry and the variables that may have problems, which will make the evaluation results not objective and accurate, cannot give specific and effective suggestions to improve the efficiency level, and lacks practical significance. To solve this problem, Färe and Grosskopf proposed a two-stage DEA model in 2000 and introduced the concept of Network DEA for the first time [24]. Tone and Tsutsui (2009) proposed a Network SBM (NSBM) model based on the weighted SBM method and conducted research on American integrated power companies. The main reason for the improvement of power system efficiency is the efficiency of each sub-sector level [25]. Cantor et al. (2020) further explored the application and significance of the NSBM model in efficiency measurement through the study of 10 power companies [26]. Therefore, this article aims to open the “black box” of the power industry, study the relationship between power production and the efficiency of the transmission and distribution sector, and use the network data envelopment analysis method based on slack variables and measurement of the efficiency of the overall power system, the power generation sector, and the transmission and distribution network sector.

In summary, given that the power industry is a complex interconnected system, the power generation industry and the transmission and distribution network both occupy a dominant position in the process of China’s power marketization, and the transmission and distribution services are at a key position in the power industry chain. Electric energy is transmitted from generation to end consumption, and the amount of electricity generated in the power generation link is used as the link between the two links. This article first adopts long-term panel data of 30 provinces and cities in China from 1998 to 2019; the time span is long and covers two major power market reform experiences, which makes up for the limitations and incompleteness of the previous research cycle. Secondly, we comprehensively consider the chain links of power generation, transmission, distribution, and power sales in the power system and their internal interactions, using the NSBM model to analyze the environment efficiency from the perspective of regional differences from the overall power industry, power generation links, and transmission and distribution network links. The research can not only improve the efficiency level of China’s power industry and narrow the gap between regions but also provide corresponding policy recommendations for optimizing the reform of the power system.

## 3. Research Method and Index System

### 3.1. Research Method

The network DEA model based on the SBM method, that is, the NSBM model, can be directly used to deal with the input redundancy and output shortage problems under the network structure of the power system. On the one hand, it has the advantages of the SBM model and incorporates the slack variable into the objective function. On the other hand, combined with the network model, it solves the shortcoming of the traditional model that treats the production process as a whole and performs “black box” processing. It can study the efficiency of different links and between links, which is helpful to reveal the internal efficiency of the system and its impact on the overall power industry. 

Suppose there are *n* homogeneous decision-making units $DMU_{j}\left(j=1,\ldots ,n\right)$, and the internal structure of each decision-making unit is K continuous departments. $x_{ijk}$ represents the input i of department k of decision-making unit $DMU_{j}$. $y_{rjk}$ represents the output r of department k of decision-making unit $DMU_{j}$. $z_{j\left(k,h\right)}{}_{l}$ represents the connection variable of department k and department h. $m_{k}$ represents the number of input types of department k. $r_{k}$ represents the number of output types of department k. Therefore, under the condition of variable returns to scale, the possible set of production is defined as $\left\{\left(X_{k},Y_{k},Z_{\left(k,h\right)}\right)\right\}$.

(1) $$\begin{array}{ll} X_{k}\geq \sum_{j=1}^{n}X_{jk}\lambda_{jk}\;\left(k=1,\ldots ,K\right), & \\ Y_{k}\leq \sum_{j=1}^{n}Y_{jk}\lambda_{jk}\;\left(k=1,\ldots ,K\right), & \\ Z_{\left(k,h\right)}=\sum_{j=1}^{n}Z_{j\left(k,h\right)}\lambda_{jk}\;\left(\forall \left(k,h\right)\right), & \\ Z_{\left(k,h\right)}=\sum_{j=1}^{n}Z_{j\left(k,h\right)}\lambda_{jh}\;\left(\forall \left(k,h\right)\right), & \\ \sum_{j=1}^{n}\lambda_{jk}=1\left(\forall k\right)\;,\;\lambda_{jk}\geq 0\;\left(\forall j,k\right), & \end{array}$$

Among them, $\lambda_{jk}$ represents the weight of the department k of the decision-making unit $DMU_{j}$. If the constraint conditions $\sum_{j=1}^{n}\lambda_{jk}=1\;\left(\forall k\right)$ are deleted, the production situation with constant returns to scale can be dealt with.

The decision-making unit to be evaluated can be expressed as:

(2) $$\begin{array}{l} X_{ok}=X_{k}\lambda_{k}+S_{k}^{-}\;\left(k=1,\ldots ,K\right),\\ Y_{ok}=Y_{k}\lambda_{k}-S_{k}^{+}\;\left(k=1,\ldots ,K\right),\\ e\lambda_{k}=1\;\left(k=1,\ldots ,K\right),\\ \lambda_{k}\geq 0,\;S_{k}^{+}\geq 0,\;S_{k}^{-}\geq 0,\;\left(\forall k\right), \end{array}$$

Among them:

(3) $$\begin{array}{ll} X_{k}=\left(X_{1k},\ldots ,X_{nk}\right)\in R^{m_{k}\times n}, & \\ Y_{k}=\left(Y_{1k},\ldots ,Y_{nk}\right)\in R^{r_{k}\times n}, & \end{array}$$

$S_{k}^{-},S_{k}^{+}$ are the slack variables of input and output.

In the NSBM model, there are many forms of connection variables. Generally, the connection variables between two adjacent departments are divided into the following two types:

(1) “Free” connection variable. The value of this variable can be adjusted arbitrarily, and the value of the connection variable is variable, that is, whether its change can be determined by neighboring departments through negotiation, the expression is:

(4) $$\sum_{j=1}^{n}\lambda_{jk}z_{j{\left(k,h\right)}_{l}}=\sum_{j=1}^{n}\lambda_{jh}z_{j{\left(k,h\right)}_{l}},\;{\left(k,h\right)}_{l}=1,\ldots ,linkfree_{\left(k,h\right)}$$

(2) “Fixed” connection variables. The value of this variable cannot be adjusted arbitrarily. The value of the connection variable is fixed. Whether it changes or not is not affected by two adjacent departments. The expression is:

(5) $$\begin{array}{l} \sum_{j=1}^{n}\lambda_{jk}z_{j{\left(k,h\right)}_{l}}=z_{o(k,h)l},\;{\left(k,h\right)}_{l}=1,\ldots ,linkfix_{\left(k,h\right)},\\ \sum_{j=1}^{n}\lambda_{jh}z_{j{\left(k,h\right)}_{l}}=z_{o(k,h)l},\;{\left(k,h\right)}_{l}=1,\ldots ,linkfix_{\left(k,h\right)} \end{array}$$

Based on the basic structure of the NSBM model, first of all, combined with the basic national conditions and status quo of China’s power system, the transmission and distribution network sectors are not separated, and only the power generation and transmission and distribution network sectors are considered. Secondly, most of the previous studies have chosen input-oriented or output-oriented models, aiming to achieve the minimum input with a certain output or to achieve the maximum output with a certain input. However, this article considers that policy makers can not only control the various inputs of the power system but can also formulate policy-regulated power demand, that is, power consumption, so the non-oriented or passive model is selected. Meanwhile, the non-oriented model can avoid the efficiency estimation bias under the interaction of multiple sectors, which can more objectively and accurately assess the environmental efficiency of China’s power industry and its evolution trend. In summary, the following passive NSBM model, including undesired output, is established:

(6) $$\begin{array}{c} \min\frac{\sum_{k=1}^{k}w^{k}\left[1-\frac{1}{m_{k}}\left(\sum_{i=1}^{m_{k}}\frac{s_{iok}^{-}}{x_{iok}}\right)\right]}{\sum_{k=1}^{k}w^{k}\left[1+\frac{1}{r_{k}+f_{k}}\left(\sum_{r=1}^{r_{k}}\frac{s_{rok}^{+}}{y_{rok}}+\sum_{r=1}^{f_{k}}\frac{u_{pok}^{+}}{b_{pok}^{t}}\right)\right]}\\ s.t.\left\{ \begin{array}{l} \sum_{j=1}^{n}\lambda_{jk}x_{ijk}+s_{iok}^{-}=x_{iok},i=1,\ldots ,m_{k},\\ \sum_{j=1}^{n}\lambda_{jk}y_{rjk}+s_{rok}^{+}=y_{rok},r=1,\ldots ,r_{k},\\ \sum_{j=1}^{n}\lambda_{jk}b_{pjk}+u_{pok}^{+}=b_{pok},p=1,\ldots ,f_{k},\\ \sum_{j=1}^{n}\lambda_{jA}z_{j{\left(A,B\right)}_{l}}=\sum_{j=1}^{n}\lambda_{jB}z_{j{\left(A,B\right)}_{l}},{\left(A,B\right)}_{l}=1,\ldots ,linkfree_{\left(A,B\right)},\\ \sum_{j=1}^{n}\lambda_{jk}=1,\\ \lambda_{jk}\geq 0,s_{iok}^{-}\geq 0,s_{rok}^{+}\geq 0,u_{pok}^{+}\geq 0,\forall i,\forall r,\forall p,\forall j,\forall k, \end{array}\right. \end{array}$$

Among them, *A* and *B* represent the power generation department and the transmission and distribution network department, respectively.

The corresponding department efficiency is:

(7) $$\rho_{k}=\frac{1-\frac{1}{m_{k}}\left(\sum_{i=1}^{m_{k}}\frac{s_{iok}^{-}}{x_{iok}}\right)}{1+\frac{1}{r_{k}+f_{k}}\left(\sum_{r=1}^{r_{k}}\frac{s_{rok}^{+}}{y_{rok}}+\sum_{r=1}^{f_{k}}\frac{u_{pok}^{+}}{b_{pok}^{t}}\right)}$$

where *k* represents the department. When *k* is *A*, it is the efficiency of the power generation department; when *k* is *B*, it is the efficiency of the transmission and distribution grid department.

### 3.2. Index System Construction and Data Sources

**Figure 1:**
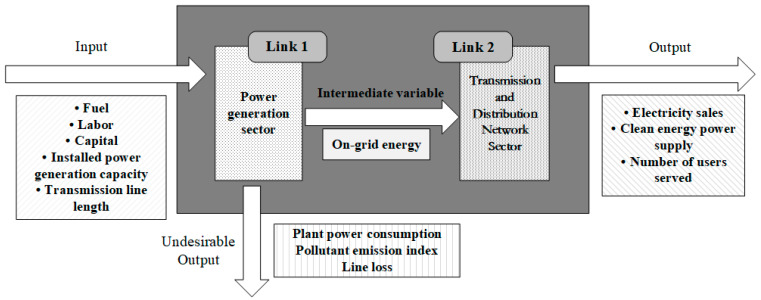
Power system production structure diagram.

The characteristic of power industry production is that production, transmission, and consumption are completed at the same time. The power generation, transmission, and use of electricity must be balanced. When examining the efficiency of the power system, the interaction of multiple sectors must be taken into consideration. Because China’s power industry currently implements an operation and management system that separates production and transmission and distribution and integrates transmission and distribution, this article constructs a vertical production structure diagram from the two departments of power generation and transmission and distribution network, as shown in Figure 1. Power generation companies use capital, equipment, personnel, fuel, etc., to generate electricity. The generated electricity is output and delivered to the power consumption terminal through the supply link. The output power is the input of the transmission and distribution department and uses it as an intermediate connection variable. Through this correlation, the output efficiency of the power generation department determines the input of the transmission and distribution department, and the transmission capacity of the transmission and distribution department determines how much electricity the power generation department needs to generate. If the power transmission capacity is limited, it will produce “whole power” in the power generation sector, resulting in idle power generation equipment, thereby affecting the efficiency of power generation. Whether the two are coordinated in development will affect the overall efficiency of the power system.

In view of the complexity of the power system and the basic national conditions for the development of power marketization as well as the actual supply process in the power production system, this paper selects physical production factors, such as the number of employees, coal consumption for power generation, investment in fixed assets, installed power generation capacity, and length of transmission lines, from the four perspectives of labor, fuel, capital, and equipment as inputs for the two sectors, as shown in Table 1.

Among them, for undesired output, there will be line loss in the entire power system, but more of it exists in the transmission and distribution network, so the line loss is regarded as the undesired output of the transmission and distribution network department. In order to reflect the development goals of energy conservation and emission reduction in the power industry, the pollutant emission index is regarded as an undesired output of environmental efficiency, and five pollutant indicators, such as carbon dioxide, sulfur dioxide, soot, waste water, and solid waste, are mainly selected.

As there is no direct environmental pollutant emission data from the power industry, the existing pollutant data come from industry or the entire industry. In order to avoid too large data discrepancies affecting the final efficiency results, first convert the existing data into data under the power industry, that is, perform the following processing of pollutants; secondly, use the entropy method to combine the five indicators into a pollutant emission index.

(8) EPE=RPE×(SCC/REC)

Among them, EPE is the electricity pollution emissions in various regions; RPE is the regional pollutant emissions; SCC is the standard coal consumption for regional power generation; REC is the regional energy consumption standard coal.

The data of the above variables are derived from the “China Electric Power Statistical Yearbook”, “China Statistical Yearbook”, “China Energy Statistical Yearbook”, “China Environment Statistical Yearbook”, China Information Bank-China Statistics Database from 1998 to 2019 in 30 provinces and cities in China, Statistical yearbooks of various provinces and cities, etc. Due to the incompleteness of the Tibet data, they were removed from the data set.

## 4. Empirical Result Analysis

### 4.1. Comprehensive Level Results of Environmental Efficiency in the Power Industry

**Figure 2:**
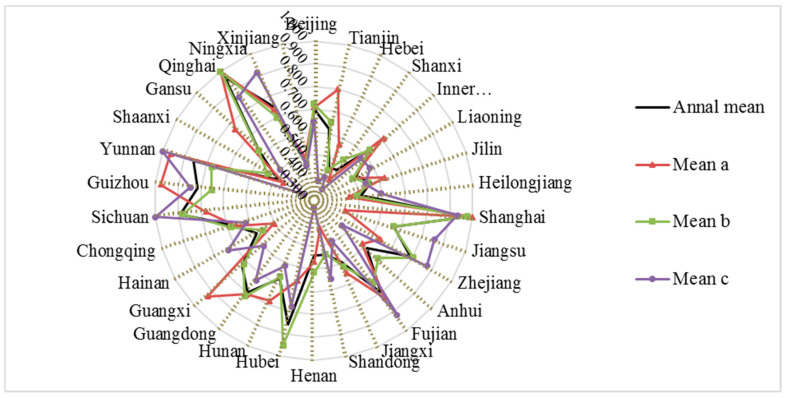
Annual average values of comprehensive level of environmental efficiency in power industry in China’s 30 provinces under different power reforms.

Based on the network SBM model considering undesired output under the variable return assumption, with the help of MaxDEA software, the overall industry and the efficiency levels of the two links in the 30 provinces and municipalities of China in different years from 1998 to 2019 were calculated. So as to compare and analyze the reasons for the difference in efficiency in different years and under different power reform policies, the details are shown in Table 2 and Figure 2.

Judging from the overall comprehensive level of environmental efficiency of the power industry in China’s 30 provinces from 1998 to 2019, the overall level of most provinces and cities is not high: the average value is around 0.672, which is far less than 1. In general, the DEA has not been able to be effective, and there is still room for improvement. It can be seen from Table 2 that the efficiency levels between provinces show significant differences. The areas with a relatively high comprehensive level of environmental efficiency in the power industry are mainly concentrated in the economically developed eastern or coastal areas and areas with superior resource endowments, such as Shanghai, Fujian, Hubei, Guangdong, Zhejiang, Yunnan, and Qinghai, etc. Among them, Shanghai has the highest level of efficiency, and the annual average value of environmental efficiency is as high as 0.971; the optimal allocation of resources has been achieved in most years (the efficiency value is 1). On the one hand, it makes full use of the optimal utilization of existing resources and technologies, and on the other hand, it also minimizes environmental pollution. However, Hebei, Shanxi, Shaanxi, Xinjiang, Liaoning, Jilin, Heilongjiang, Henan, Shandong, and other regions have lower efficiency levels (<0.6), which may be caused by excessive emissions, abuse of resources, and imperfect related technical equipment in the development of coal. Among them, Shanxi, Hebei, Shaanxi, Henan, and other places are also used as centralized power output areas, and their environmental efficiency is often low, reflecting the phenomenon of “resource curse” and “polluted paradise” at the regional level.

Under the influence of different power policy reforms, there are still big differences in the overall efficiency level of various provinces in China, including the “separation of government and enterprises” in 1998, the “separation of power plants and grids and bidding on the Internet” in document No. 5 in 2002, and the “control the middle and open the two ends” power reform in document No. 9 in 2015. It can be seen from Figure 2 that the effects of the three reforms on provinces and cities are quite different. Among them, the environmental efficiency of Shanghai, Zhejiang, Fujian, Hubei, Sichuan, Yunnan, Qinghai, and other places have been improved to varying degrees under the influence of the power reform policy; it shows that these cities can effectively adapt to and obey the direction of the national power reform and find a suitable development direction for the province and city from it. The power system reform in 1998 aimed to separate the government from the enterprise, break the monopoly, and adjust the allocation of power resources through market means rather than administrative monopoly means, effectively regulate the development of the power market, and improve the efficiency level of many cities. The power reform in 2002 focused on “separation of power plants and grids and bidding on the Internet”, which aimed to break monopoly, introduce competition, improve efficiency, reduce costs, optimize resource allocation, and promote power development. To a certain extent, it failed to take the environment as a major development goal into consideration, so the environmental efficiency of most provinces in the country is not high. Compared with the three reforms, the power reform in 2015 effectively promoted the development of eight provinces and cities, including Jiangsu, Zhejiang, Shandong, Fujian, Hainan, Sichuan, Yunnan, and Ningxia; it failed to effectively improve the environmental efficiency of 12 provinces and cities, including Chongqing, Guangxi, Guangdong, Hunan, Hubei, Henan, Jiangxi, Anhui, Inner Mongolia, Shanxi, Hebei, and Tianjin. Most of these provinces are power output areas or regions rich in energy resources, which may have undertaken more pressures of resource and environmental compensation or failed to effectively and rationally use existing resources.

### 4.2. Results of Sub-Link Efficiency of Environmental Efficiency in Power Industry

**Figure 3:**
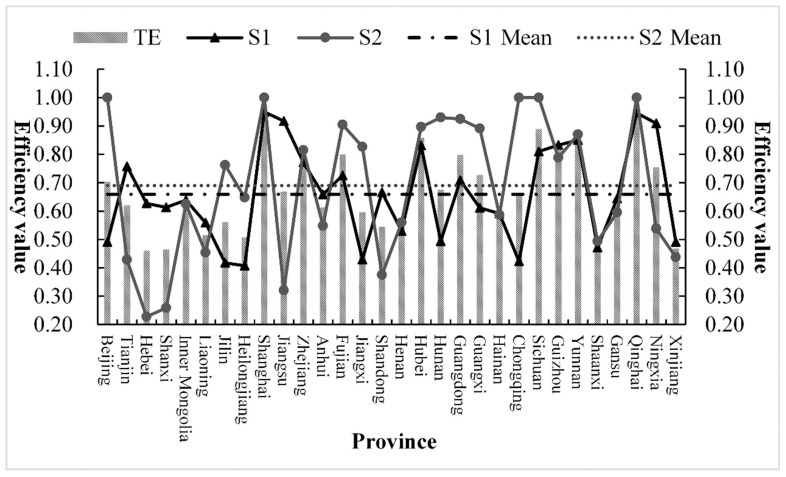
Comparative trend of environmental efficiency of power industry in 30 Provinces in China. Note: TE represents the overall efficiency; S1 represents the power generation efficiency of the first link; S2 represents the power transmission and distribution network efficiency of the second link.

**Figure 4:**
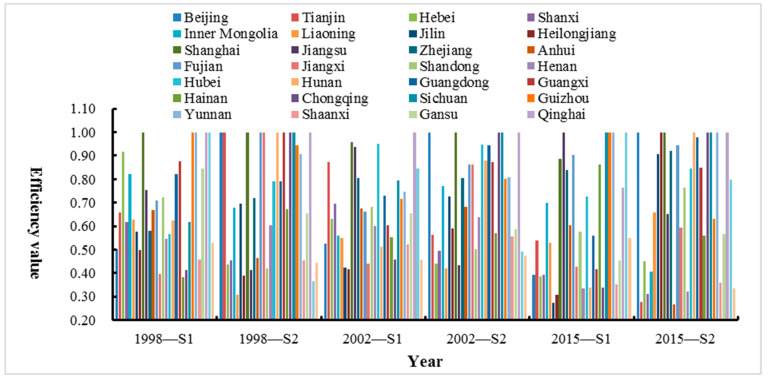
The environmental efficiency level of the power industry in China’s 30 provinces under different power reforms.

In order to explore the main reasons for the differences in the comprehensive level of environmental efficiency between provinces and cities, we must keep in mind that the power industry is a complex interconnected system, which mainly includes power generation, transmission, distribution, and sales, and the overall efficiency level is affected by multiple links. Therefore, this article is mainly based on the basic national conditions of China’s power industry and does not separate power transmission and distribution; the effects of power generation and transmission and distribution on the overall efficiency are discussed, and the specific changes are shown in Table 3 and Figure 3 and Figure 4.

From the perspective of sub-link efficiency, in general, the power generation efficiency of the first link in most provinces and municipalities is higher than the transmission and distribution efficiency of the second link; this shows that the low transmission and distribution efficiency is the main reason for the low comprehensive level of efficiency in the power industry. However, it can be seen from Figure 3 that the low efficiency of some provinces, such as Jilin, Fujian, Jiangxi, Chongqing, and Sichuan, during the study period is mainly caused by the low efficiency of the power generation. It shows that these provinces have advantages in the management mode and means of the transmission and distribution link, and it is urgent to improve the technical level and resource allocation of the power generation link so that the two links can be better coordinated and the optimal allocation can be realized.

It can be seen that the reasons leading to low efficiency level are complex and diverse, which are not only affected by the differences of provinces themselves but also affected by the external environment and relevant policies, specifically reflected in geographical location, economic development, technological level, management mode, local policies, and so on.

During the research period, only two regions, Shanghai and Qinghai, met the two links to achieve DEA effectiveness at the same time, indicating that during 1998–2019, most provinces and municipalities failed to achieve the coordinated development of energy power, environment, and economy. The power generation efficiency of Tianjin, Hebei, Shanxi, Inner Mongolia, Jiangsu, Zhejiang, Anhui, Henan, Shaanxi, Ningxia, and Xinjiang is higher than the transmission and distribution efficiency, the efficiency value is mostly less than 1, and DEA is not effective. However, these areas are mostly rich in coal resources and concentrated power output. There is a certain degree of “resource curse” phenomenon and the development between power generation and transmission and distribution links is still not coordinated. In the future, we should focus on improving the efficiency of transmission and distribution. The power generation efficiency in Beijing, Jilin, Fujian, Jiangxi, Hubei, Hunan, Chongqing, Sichuan, Guangxi, Yunnan, and other regions is lower than the transmission and distribution efficiency, and these regions have relatively good power transmission and distribution capabilities, especially Beijing and Fujian, which have achieved the most efficient level in most years but continue to improve the power generation efficiency level in each region. The power generation efficiency of Liaoning, Heilongjiang, Shandong, Guangdong, Gansu, and other places was higher than the transmission and distribution efficiency at the beginning, after continuous policy reforms and improvements in management, technology, etc. The efficiency of transmission and distribution keeps catching up or even surpassing the level of power generation efficiency, achieving a gradual increase in the overall efficiency level. The transmission and distribution efficiency of Hainan, Guizhou, and other places is higher than the power generation efficiency in the early years; later, due to backward power generation technology and equipment and uncoordinated management mechanisms, the opposite development trend appeared. On the whole, the undertaking and development of the two links in various provinces, municipalities, and districts have not achieved the optimal allocation; there is a considerable degree of imbalance between power supply and demand and power waste.

Through the above analysis, under the effects of different power reforms, the environmental efficiency of the power industry between provinces and cities is quite different in the two links of power generation and transmission and distribution. It is necessary to study the reasons for these differences, as shown in Figure 4. The separation of government and enterprise in 1998 effectively promoted the efficiency of power generation in Shanghai, Guizhou, Yunnan, Qinghai, and Ningxia and the efficiency of transmission and distribution in Beijing, Tianjin, Shanghai, Fujian, Jiangxi, Hunan, Guangxi, Chongqing, Sichuan, and Qinghai. On the whole, the power reform in 1998 improved the transmission and distribution capabilities of some provinces and cities. Among them, Shanghai and Qinghai both achieved DEA effectiveness in two links. Although the power system reform of “separation of power plants and grids” in 2002 promoted the improvement of power generation efficiency and transmission and distribution efficiency in most provinces and municipalities, there are still not many provinces that achieve the best efficiency, only the transmission and distribution efficiency of Beijing, Shanghai, Chongqing, Sichuan, and Qinghai reached 1; therefore, the power generation efficiency has not been effectively improved under this reform. In 2015, the power market-oriented reform of “control the middle and open the two ends” effectively regulated the transmission and distribution links, and more provinces and municipalities have reached the most effective state of DEA. In comparison, only Jiangsu, Sichuan, Guizhou, Yunnan, and Ningxia have the power generation efficiency of 1; thus, the power generation efficiency level of most other provinces and cities still needs further improvement. The reasons are as follows: first, there are redundant inputs in various resources, equipment, personnel, fuel, capital, etc., in the power generation process and the backwardness of related technologies, which causes a great deal of waste and environmental pollution; second, the competition mechanism, electricity price reform, and resource price compensation in the power generation market still need to be adapted to local conditions.

### 4.3. Comprehensive Comparative Analysis of Regional Differences in Environmental Efficiency of Power Industry

In order to further explore the regional differences in the environmental efficiency of China’s power industry, we must point out that according to the division standards of regional power grids, China is divided into six regional power grids: northeast, north China, central China, eastern China, south China and northwest, as shown in Table 4.

**Figure 5:**
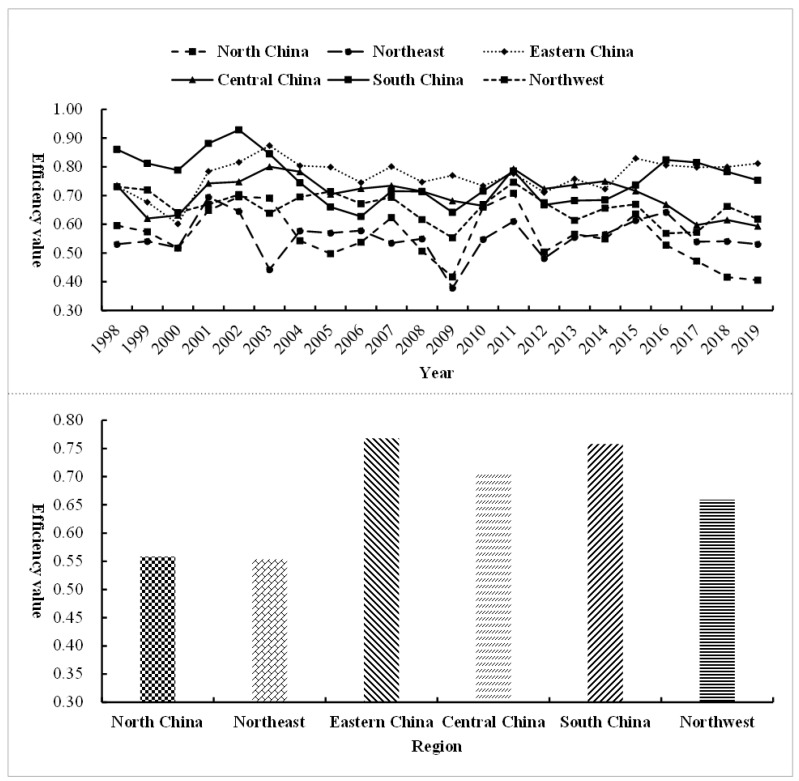
Comparison of the comprehensive level of environmental efficiency in six regions of China.

From a regional perspective, from 1998 to 2001, the overall environmental efficiency of each region showed a “V”-shaped trend. From 2002 to 2014, there was a trend of volatility and twists; from 2015 to 2019, the overall environmental efficiency level was at a relatively high level, and north China and northeast showed a downward trend, as shown in Figure 5.

The main reasons for the above changes are as follows: in 2004, the electric power industry implemented a series of environmental protection policies, such as “scale the large and suppress the small” and desulfurization and denitrification”, which promoted the management and technical level of power enterprises and then affected the environmental efficiency to varying degrees. Due to the global economic crisis at the beginning of 2007 and the downward pressure on the global economy in 2012, the environmental efficiency of the power industry showed a downward trend. Although the reform goal of “Circular No. 5” of electric power was to improve efficiency, optimize resource allocation, and promote power development, domestic enterprises were in a shrinking market, and the efficiency improvement of the power generation link and the transmission and distribution network link was limited. Therefore, the development of the power industry and the improvement of the management level of power enterprises had been affected to a considerable extent, and a healthy development of a coordinated and orderly power market system had not been constructed. The new round of power system reform in 2015 relatively effectively promoted the improvement of environmental efficiency in most regions. However, due to unreasonable internal structure and resource allocation, some regions could not adapt to the effects of reform policies.

From 1998 to 2019, the overall change trend of the comprehensive level of environmental efficiency of the six regional power industries is roughly the same. However, the regional differences are obvious; from high to low, they are east China, south China, central China, northwest, north China, and northeast, showing a situation of “high in the southeast and low in the northwest”. The highest in east China is 0.768, followed by south China at 0.758 and central China at 0.704, all higher than the national average (0.672). The other three regions are all lower than the average level, indicating that the environmental efficiency values of the six regions need to be improved during the sample period. East China and south China are at a relatively high level of efficiency, mainly due to the high degree of economic development and as the input place of coal and electricity resources in the regions, which can make full use of existing resources and advanced technology and equipment, but there is still a certain distance between it and the optimal resource allocation efficiency. Central China and northwest are at a medium efficiency level, and most of the provinces are areas rich in coal or clean energy resources. Because of its backward economy, it is unable to have more advanced technology and equipment to make full use of the advantages of existing resource endowments and take on the responsibility of environmental protection that they should not have. In the future, it is still necessary to further improve the construction of the resource compensation mechanism to make up for the cost of environmental inefficiency. North China and northeast are at a relatively low level of efficiency, and the northern regions are mostly power output areas and old industrial bases in the northeast. Due to the outdated technical equipment and backward management concepts, their environmental efficiency is low, which needs to strengthen for improvement in the future.

### 4.4. Sub-Link Comparative Analysis of Regional Differences in Environmental Efficiency of the Power Industry

**Figure 6:**
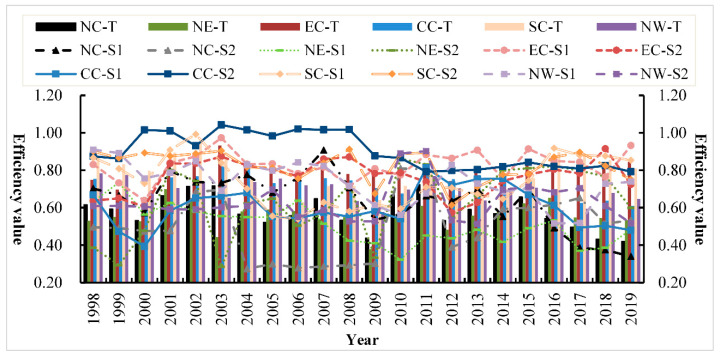
Comparative trend chart of regional differences in environmental efficiency in China’s power industry. Note: NC, north China; NE, northeast; EC, eastern China; CC, central China; SC, south China; NW, northwest.

**Figure 7:**
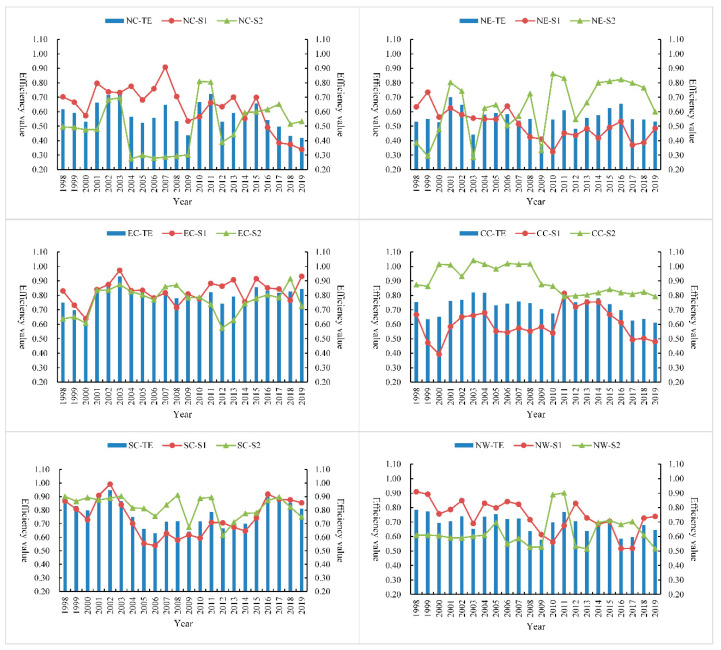
Comparative trend chart of sub-link efficiency for regional differences in environmental efficiency of China’s power industry.

In order to further analyze the differences in efficiency levels among different regions, it will be elaborated from the perspective of annual links, as shown in Figure 6 and Figure 7. The variation trend of the annual efficiency level in each region is complex and not regular, indicating that the developmental course of China’s power industry is extraordinary, and progress is made in stability as a whole. As can be seen from the complex trend in Figure 7, the improvement of environmental efficiency in the power industry is necessary and needs to be adjusted to local conditions.

Analysis of annual regional differences. For north China, the power generation efficiency (0.635) was higher than the power transmission and distribution network efficiency (0.500) in most years and showed an opposite trend after 2015, indicating that the power reform in 2015 played a positive role in the region. For northeast China, the efficiency levels of the two links showed an irregular trend of change, but the efficiency of transmission and distribution network was higher than that of power generation after 2009 and showed an inverted “U”-shaped trend from 2012 to 2019 and reached the maximum value in 2016, indicating that the policy effect of the power reform in 2015 had a lag. For eastern China, the changes of power generation efficiency and transmission and distribution network efficiency are similar, both at a high efficiency level, with efficiency values of 0.825 and 0.765, respectively. Efficiency level in the six regions in the first place, assuming the “leader” and radiation driving role. For central China, except for 2011, the efficiency of power transmission and distribution network (0.901) was higher than that of power generation (0.603) in other years. After 2011, the efficiency gap became smaller, and the power generation efficiency was lower than the comprehensive efficiency level (0.727), indicating that the low power generation efficiency in this region was mainly the cause of the low power generation efficiency. For south China, the efficiency of power transmission and distribution network (0.825) is higher than that of power generation (0.744) in most years, showing a “U”-shaped trend of overall change, ranking second among the six regions. The power reform in 2015 obviously promoted the efficiency level of the region, with the average comprehensive efficiency of 0.862 after 2015, higher than the annual average of 0.777. For northwest China, the efficiency trend is similar to that of north China. The power generation efficiency of most provinces (0.736) is higher than that of power transmission and distribution network (0.630). After 2009, the efficiency levels of the two links show a trend of change. It shows that the development of electric power industry in this region is in the exploratory stage, and the appropriate coordination mechanism and development path have not been found. In the future, according to the unique geographical and resource endowment advantages of this region, we can imitate and learn the development mode of high-efficiency region and strive to find a suitable operation mechanism of “management mode + technical means + resource endowment”.

### 4.5. Analysis on the Improvement Path of Environmental Efficiency in the Power Industry

According to the above analysis, from 1998 to 2019, most cities have varying degrees of input-output redundancy. That is, the input is too high, or the output is insufficient; DEA is relatively ineffective, resulting in its environmental efficiency, making it unable to reach optimal improvement. Due to the limitations of space, this article only takes 2019 as an example to carry out redundancy analysis on the input-output indicators of environmental efficiency of the power industry in 30 provinces in China. Moreover, on this basis, we point out the path and direction of the next efficiency improvement for each province and city, as shown in Table 5.

Among the 30 provinces and municipalities in 2019, in addition to Beijing, Shanghai, Jiangsu, Guangdong, Hainan, Sichuan, Yunnan, and Qinghai, the remaining 22 regions are relatively ineffective in DEA, mainly distributed in north China, northeast, central China, and northwest. As can be seen from Table 5, the regions of DEA relatively ineffective have varying degrees of redundancy in terms of the number of power employees, coal consumption for power generation, power investment in fixed assets, power generation installed capacity, and the length of transmission lines. In addition, some provinces and cities have serious environmental pollution, factory power consumption, and line losses.

Therefore, this article focuses on a few typical areas and proposes improvement paths: (1) Tianjin, Hebei, Shanxi, and Shandong in north China have low overall environmental efficiency due to coal consumption for power generation, fixed asset investment, and excessive consumption of transmission lines. In the future, technological improvement should be accelerated to reduce coal consumption for power generation and optimize the transmission capacity of power transmission lines and reasonably allocate capital investment in the power industry. (2) Inner Mongolia, Liaoning, Jilin, and Heilongjiang in the northeast region have low environmental efficiency due to the five aspects of the number of power employees, coal consumption for power generation, power asset investment, power generation installed capacity, and serious consumption of transmission lines. Next, they should focus on accelerating the adjustment of industrial structure, reducing the total energy consumption, optimizing the deployment of electric power workers, improving the performance of outdated power generation and transmission equipment, encouraging the development of green ecological industries, and gradually replacing traditional old industrial industries. (3) Jiangxi, Henan, Hubei, Hunan, and Chongqing in central China are severely redundant due to the number of power workers, coal consumption for power generation, power asset investment, and transmission line redundancy, which affect the improvement of environmental efficiency. Among them, Henan, as a power output place, has a better performance in the configuration of transmission and distribution lines than other regions; all regions should strengthen technological innovation and optimize the allocation of manpower and energy resources in the future. (4) Shaanxi, Gansu, Ningxia, and Xinjiang in the northwest region have suffered serious consumption of coal for power generation, power generation installed capacity, and transmission lines, which have become the three main factors restricting the improvement of environmental efficiency. There is huge potential for improvement in energy saving and environmental protection. The provinces and municipalities in this region should give full play to their natural advantages and develop more renewable clean energy to replace the consumption of traditional energy. At the same time, these regions should introduce advanced equipment and technology, strengthen mutual exchanges and cooperation between the east and west regions for mutual benefit, and optimize the utilization rate of resource integration. 

## 5. Conclusions and Suggestion

This paper uses a two-link NSBM model to measure the environmental efficiency of the power industry in 30 provinces and municipalities in China from 1998 to 2019. One is to make up for the “black box” evaluation shortcomings of the traditional DEA model, and the other is to consider the environmental indicators of undesired output, making the measured environmental efficiency more referential.

Our research indicates that (1) from 1998 to 2019, the overall environmental efficiency level (0.672) of 30 provinces in China is not high. In addition, the differences between provinces and cities are large, and the DEA has not been effective in general. With the implementation of the three power reforms, the power marketization reform in 2015 effectively promoted the improvement of environmental efficiency. In general, it will gradually improve in a favorable direction. (2) In terms of efficiency in different links, the environmental efficiency of the power industry between provinces and cities under different power reforms is quite different in the two links of power generation and transmission and distribution: the power generation efficiency of the first link is higher than the transmission and distribution efficiency of the second link in the most provinces and municipalities. The low transmission and distribution efficiency is the main reason for the low comprehensive level of environmental efficiency. (3) From a regional perspective, the overall change trend of environmental efficiency in the six regions is roughly the same, with obvious regional differences; from high to low, they are east China (0.768), south China (0.758), central China (0.704), northwest (0.659), north China (0.559), and northeast (0.554), showing a situation of “high in the southeast and low in the northwest”. (4) Due to differences in economic development and natural resource endowments in different provinces and cities in each region, there are varying degrees of redundancy in five aspects, including the number of power employees, power generation coal consumption, power asset investment, power generation installed capacity, and transmission line length, which is the important reason for the low environmental efficiency of the power industry.

Based on the above research conclusions and the production characteristics of the power system, the aim is to improve the environmental efficiency of various provinces and cities and promote sustainable and healthy development. This article gives suggestions from the three perspectives of the power industry, power generation, and transmission and distribution network links.

(1) For the entire power industry: First, the exchange and cooperation of electric power technology between regions should be promoted. Although there is a “catch-up effect” in the power environmental efficiency between regions, there is a large gap between regions, which can be communicated through management concepts. High-efficiency Shanghai and Qinghai should give full play to their radiating and leading role, and low-efficiency provinces should learn and refer to the development experience of high-efficiency provinces, for example, learning professionals’ “climate-environmental” ability [27] and narrowing the regional gap. Second, these regions should continue to deepen the reform of the power system and improve the efficiency of power environment. The power system reform effect in 2015 was better than 2002 and 1998, but they were not able to increase the efficiency level greatly, which may be affected by the lag and imperfections of policy reforms. In the future, it should continue to intensify and promote reforms in deep-level system reforms, such as the separation of transmission and distribution, the setting of electricity prices and the power market, promotion of fair competition, and coordinated development of the power industry, enhancement of competitive vitality, and improvement of efficiency. Third, they should promote technical exchanges and cooperation between regions and coordinate the coordinated development of all regions. Through technical cooperation and integration between the eastern and western regions, the provinces in the western and northern regions can be driven to optimize resource allocation, reduce unnecessary consumption of coal for power generation, promote the optimization and upgrading of industrial structure, and constantly narrow the efficiency gap between regions. For example, national technical exchange and integration meetings should be held regularly. East China and south China, as typical representatives, should share and teach the management mode, technical means, how to solve problems, and how to effectively use and dispose clean fuels in the power industry in the region so as to give full play to their role as the leader of the industry efficiency level and advice. However, the northwest, north China, northeast, and other regions should timely and truthfully put forward the shortcomings and existing problems of the development of the electric power industry in each region and strive to find a way to solve the problems in the conference. In the future, the focus should be based on the power demand of each region and its own power generation capacity and on rationally allocating resources through market mechanisms, form an effective power trade chain.

(2) For the power generation link: First, clean and efficient power production technology should be developed to reduce pollution emissions and the power consumption of the power plants themselves, optimize the new energy power generation subsidy policy, and promote a better and more sustainable power supply structure. The proportion of new energy generation in China’s provinces is generally low, with an average of 26.91% from 1998 to 2019; there is still a large space for improvement. Qinghai, Hubei, Sichuan, Yunnan, Guangxi, and other places have a relatively high proportion of new energy power generation, all reaching more than 50%. Moreover, the environmental technical efficiency of power generation in each province is at a relatively high level, and its high proportion of clean energy power generation structure, suitable production technology, and reasonable resource allocation can provide a better leading and driving role for other provinces and cities. At the same time, there should be focus on accelerating the promotion of new energy subsidy policies, increasing the proportion of new energy power generation, and finally forming a clean power supply structure. Second, it is important to strengthen the regulation and supervision of energy saving and emission reduction in areas rich in coal resources and establish a reasonable inter-regional resource consumption ratio and pollution compensation mechanism. Some provinces with low environmental efficiency in power generation are concentrated in areas with abundant coal resources; it not only shows that these areas are under-utilizing existing resources and insufficient environmental governance, etc., but also that it is necessary to strengthen energy saving and emission reduction regulations. It also reflects the phenomenon of “resource curse” and “pollution paradise” at the regional level, and a complete pollution compensation mechanism needs to be established to increase the enthusiasm of power generation companies to protect the environment.

(3) For the transmission and distribution network link: First, the proportion of transmission technology applications in UHV grids should be increased; the construction of large-scale, long-distance and high-efficiency transmission line channels should be strengthened, the allocation of power transmission and distribution network resources should be optimized, and the reasonable distribution of power transmission and distribution and economic benefits among regions should be realized. The geographical distribution of China’s energy bases and load centers is very uneven and far away, with 76–80% of coal power, hydropower, wind power, and other bases mostly concentrated in the north, northwest, and southwest regions. In addition, more than 70% of the energy demand is mainly concentrated in the economically developed areas in the east and middle. Based on the characteristics of energy and demand distribution, it is particularly important to strengthen China’s strategic overall power grid planning and optimization. On the other hand, in fact, due to factors such as technology and funding, the implementation of China’s power grid planning is not high; notably, the construction of the UHV power grid transmission channel from the energy base to the load center is lagging behind, and the trans-regional transmission capacity is insufficient. In the future, it is urgent to optimize and configure the transmission and distribution lines in various regions to avoid unnecessary line idle. Second, it is necessary to formulate a reasonable electricity price mechanism and resource price compensation mechanism. Although promoting the construction of UHV power grid projects can avoid the co-location of traditional electricity production and consumption, we must realize the effective separation of power generation and electricity space and enable regions with resource endowment advantages to concentrate power generation first, and implementing centralized power input measures for power consumption areas can effectively optimize the optimization and configuration of power transmission and distribution network resources. However, the imperfection and irrationality of the electricity price mechanism when regions with rich resource endowments concentrate on outputting power means that high technical efficiency has not been achieved, and there is a certain degree of “resource curse” phenomenon. Therefore, when formulating price policies, the inter-regional resource price compensation mechanism should be fully considered.

In this study, due to incomplete relevant data, we did not consider the environmental efficiency of the power industry from a micro perspective. Considering that China still implements the development mechanism of separation of power production and transmission and distribution and integration of transmission and distribution, this paper failed to separate the transmission and distribution departments of the power industry and thus failed to deeply explore the influence of the internal influence mechanism between transmission and distribution departments on the overall efficiency level. The above problems can be improved and strengthened in the future. We only studied the level of environmental efficiency in the power industry as a whole. Investigation of the validity and applicability of our results in other power industry, such as hydropower and thermal power, would be an interesting topic with more of a micro perspective. In addition, different scenarios are selected to simulate and predict the evolution trend of environmental efficiency in the power industry under different scenarios, which is also an important research direction in the future.

## Figures and Tables

**Table 1:** Construction of power system input-output indicator system.

	Variable Selection	Variable Description
Input variable	Electricity practitioner	Due to the lack of separate statistical data on the number of workers in the power industry, the number of employees in the power and heat production and supply industries, which are highly correlated, is used instead. The unit is 10,000 people (X1).
	Coal consumption for power generation	Coal consumption for power generation is fuel input. Due to the lack of data on standard coal consumption for power generation in some years (1998–2006), the product of thermal power generation and standard coal consumption for power generation is used for expression. The unit is 10,000 tons of standard coal (X2).
	Investment in fixed assets	Since there is no fixed asset investment related to electricity, the fixed asset investment of the electricity, steam, and hot water production and supply industries is selected instead. The unit is 100 million yuan (X3).
	Installed power generation capacity	Select the total installed power generation capacity of each province and city (including thermal power, hydropower, nuclear power, wind power, biomass power generation installed capacity, etc.). The unit is 10,000 KW (X4).
	Transmission line length	The loop length of overhead lines above 35 kV by region. The unit is km (X5).
Intermediate variable	On-grid energy	The total annual net power generation of thermal power, hydropower, wind power, nuclear power, and other energy sources in each region; on-grid power = power generation-plant power consumption, unit: 100 million Kwh (I).
Expected output variable	Electricity Sales	The actual amount of electricity supplied to users by the power grid in each region, in 100 million Kwh (Y1).
	Number of users served	The number of users ultimately served by the transmission and distribution of electricity in each region; the unit is household (Y2).
	Clean energy power supply	The total power generation of other energy sources (wind power, hydropower, nuclear power, biomass power generation, etc.) other than thermal power in each province and city; the unit is 100 million Kwh (Y3).
Undesired output variable	Pollutant emission index	Select five environmental pollution indicators, such as waste gas, waste water, and waste residues, and use the entropy method to process them into a pollutant emission index (U1).
	Plant power consumption	Power plant’s own electricity consumption when generating electricity, plant power consumption = power generation × plant power consumption rate; unit: 100 million Kwh (U2).
	Line loss	The amount of line loss during transportation and transmission of electricity, in units of 100 million Kwh (U3).

**Table 2:** The comprehensive level of environmental efficiency of the power industry in China’s 30 provinces of 1998–2019.

Province	1998	2001	2004	2007	2010	2013	2016	2019	Annal Mean	Mean a	Mean b	Mean c
Beijing	0.726	0.784	0.671	1.000	0.670	0.718	0.732	0.599	0.703	0.709	0.723	0.646
Tianjin	0.790	1.000	0.583	0.583	0.853	0.654	0.360	0.297	0.620	0.800	0.655	0.387
Hebei	0.641	0.597	0.468	0.435	0.470	0.494	0.418	0.380	0.461	0.571	0.445	0.413
Shanxi	0.401	0.461	0.637	0.635	0.731	0.482	0.356	0.347	0.465	0.414	0.522	0.359
Inner Mongolia	0.413	0.795	0.761	0.757	0.640	0.538	0.510	0.697	0.633	0.711	0.631	0.576
Liaoning	0.542	0.541	0.504	0.470	0.499	0.513	0.863	0.453	0.515	0.495	0.495	0.583
Jilin	0.737	0.721	0.721	0.547	0.542	0.538	0.574	0.405	0.561	0.627	0.550	0.537
Heilongjiang	0.431	0.723	0.324	0.364	0.511	0.632	0.621	0.568	0.507	0.454	0.489	0.597
Shanghai	1.000	1.000	1.000	1.000	1.000	1.000	1.000	1.000	0.971	1.000	0.976	0.933
Jiangsu	0.452	0.466	0.421	0.737	0.817	0.751	0.819	0.808	0.668	0.441	0.666	0.855
Zhejiang	0.671	0.695	0.950	0.748	0.781	0.820	0.889	0.882	0.789	0.638	0.804	0.873
Anhui	0.537	1.000	0.862	0.703	0.452	0.496	0.448	0.471	0.612	0.584	0.678	0.464
Fujian	1.000	0.760	0.785	0.817	0.619	0.722	0.871	0.901	0.800	0.830	0.745	0.921
Jiangxi	0.742	0.630	0.660	0.658	0.495	0.568	0.509	0.455	0.595	0.648	0.616	0.496
Shandong	0.420	0.399	0.354	0.463	0.573	0.481	0.771	0.405	0.545	0.424	0.540	0.654
Henan	0.429	0.752	0.720	0.716	0.534	0.515	0.303	0.338	0.542	0.569	0.616	0.329
Hubei	0.762	0.667	1.000	1.000	1.000	0.913	0.944	0.576	0.858	0.659	0.950	0.777
Hunan	1.000	0.751	0.636	0.642	0.553	0.739	0.633	0.593	0.675	0.781	0.666	0.614
Guangdong	0.844	1.000	0.843	0.893	0.732	0.762	0.825	0.599	0.798	0.808	0.820	0.733
Guangxi	1.000	1.000	0.695	0.698	0.625	0.611	0.618	0.510	0.728	0.928	0.716	0.597
Hainan	0.460	0.581	0.436	0.507	1.000	0.462	0.828	0.833	0.590	0.503	0.560	0.736
Chongqing	0.710	0.657	0.683	0.643	0.657	0.689	0.625	0.601	0.663	0.659	0.683	0.615
Sichuan	0.767	1.000	1.000	0.745	0.747	1.000	1.000	1.000	0.889	0.777	0.881	1.000
Guizhou	1.000	0.909	1.000	0.861	0.653	0.576	0.849	0.825	0.814	0.977	0.752	0.846
Yunnan	1.000	0.917	0.749	0.619	0.568	1.000	1.000	1.000	0.858	0.962	0.772	1.000
Shaanxi	0.530	0.457	0.588	0.457	0.548	0.433	0.321	0.346	0.481	0.457	0.536	0.356
Gansu	0.851	0.683	0.754	0.836	0.529	0.496	0.426	0.495	0.624	0.766	0.627	0.501
Qinghai	1.000	1.000	1.000	1.000	1.000	1.000	0.657	1.000	0.968	1.000	1.000	0.861
Ningxia	0.738	0.734	0.722	0.720	0.660	0.734	1.000	0.744	0.754	0.736	0.698	0.916
Xinjiang	0.537	0.474	0.412	0.457	0.608	0.407	0.438	0.507	0.468	0.492	0.465	0.459
Mean	0.704	0.738	0.698	0.690	0.669	0.658	0.674	0.621	0.672	0.681	0.676	0.654

Note: Due to the large number of data results, only the results of some years are listed; at the same time, for the convenience of comparison and analysis, this table lists the phased average levels under the influence of different power policy reforms: the average value is the average value of 1998–2019; the average value a is the average value of 1998–2001; the average value b is the average value of 2002–2014; the average value c is the average value of 2015–2019.

**Table 3:** The environmental efficiency level by link of the power industry in China’s 30 provinces of 1998–2019.

Province	1998		2004		2007		2010		2013		2016		2019	
	S1	S2	S1	S2	S1	S2	S1	S2	S1	S2	S1	S2	S1	S2
Beijing	0.530	1.000	0.436	1.000	1.000	1.000	0.434	1.000	0.517	1.000	0.540	1.000	0.313	1.000
Tianjin	0.640	1.000	1.000	0.570	1.000	0.559	0.748	1.000	1.000	0.271	0.515	0.242	0.324	0.359
Hebei	1.000	0.438	0.754	0.467	0.709	0.451	0.491	0.440	0.602	0.343	0.405	0.436	0.300	0.491
Shanxi	0.630	0.481	1.000	0.431	1.000	0.424	0.538	1.000	0.672	0.216	0.388	0.313	0.379	0.303
Inner Mongolia	0.642	0.392	0.591	1.000	0.583	1.000	0.382	1.000	0.696	0.316	0.501	0.523	1.000	0.274
Liaoning	0.642	0.403	0.635	0.322	0.596	0.293	0.338	0.725	0.478	0.563	1.000	0.671	0.358	0.584
Jilin	0.672	0.828	0.521	1.000	0.427	0.715	0.267	0.926	0.391	0.745	0.270	1.000	0.311	0.537
Heilongjiang	0.579	0.224	0.453	0.143	0.455	0.238	0.306	0.797	0.369	1.000	0.350	1.000	0.259	1.000
Shanghai	1.000	1.000	1.000	1.000	1.000	1.000	1.000	1.000	1.000	1.000	1.000	1.000	1.000	1.000
Jiangsu	0.769	0.486	0.707	0.420	1.000	0.369	1.000	0.562	1.000	0.403	1.000	0.567	1.000	0.539
Zhejiang	0.630	0.728	1.000	0.881	0.761	0.730	0.683	0.918	0.904	0.702	0.810	1.000	1.000	0.717
Anhui	0.677	0.340	0.763	1.000	0.491	1.000	0.599	0.247	0.764	0.121	0.588	0.253	0.603	0.286
Fujian	1.000	1.000	0.631	1.000	0.686	1.000	0.424	0.892	0.759	0.671	0.779	1.000	1.000	0.762
Jiangxi	0.557	1.000	0.416	1.000	0.413	1.000	0.330	0.726	0.554	0.588	0.390	0.677	0.410	0.519
Shandong	0.712	0.410	0.603	0.358	0.777	0.324	0.616	0.512	0.676	0.208	0.608	1.000	0.379	0.442
Henan	0.587	0.208	0.521	1.000	0.544	0.956	0.532	0.536	0.682	0.282	0.352	0.234	0.261	0.445
Hubei	0.734	0.803	1.000	1.000	1.000	1.000	1.000	1.000	1.000	0.791	1.000	0.866	0.508	0.670
Hunan	1.000	1.000	0.560	0.743	0.474	0.878	0.386	0.788	0.553	1.000	0.370	1.000	0.301	1.000
Guangdong	0.938	0.712	0.801	0.902	0.816	1.000	0.541	1.000	0.687	0.867	0.699	1.000	0.396	0.883
Guangxi	1.000	1.000	0.477	1.000	0.482	1.000	0.518	0.774	0.581	0.652	0.344	1.000	0.437	0.612
Hainan	0.324	0.650	0.417	0.464	0.537	0.465	1.000	1.000	0.457	0.470	1.000	0.587	1.000	0.599
Chongqing	0.503	1.000	0.456	1.000	0.388	1.000	0.412	1.000	0.467	1.000	0.357	1.000	0.316	1.000
Sichuan	0.600	1.000	1.000	1.000	0.563	1.000	0.566	1.000	1.000	1.000	1.000	1.000	1.000	1.000
Guizhou	1.000	1.000	1.000	1.000	0.761	1.000	0.462	0.921	0.597	0.546	1.000	0.638	1.000	0.580
Yunnan	1.000	1.000	0.797	0.683	0.546	0.720	0.443	0.743	1.000	1.000	1.000	1.000	1.000	1.000
Shaanxi	0.556	0.494	0.550	0.642	0.442	0.480	0.399	0.755	0.543	0.279	0.354	0.276	0.333	0.364
Gansu	1.000	0.644	0.850	0.621	1.000	0.607	0.442	0.650	0.466	0.539	0.315	0.582	0.490	0.501
Qinghai	1.000	1.000	1.000	1.000	1.000	1.000	1.000	1.000	1.000	1.000	0.412	1.000	1.000	1.000
Ningxia	1.000	0.371	1.000	0.333	1.000	0.329	0.418	1.000	1.000	0.361	1.000	1.000	1.000	0.387
Xinjiang	0.608	0.437	0.461	0.343	0.487	0.416	0.328	1.000	0.453	0.342	0.496	0.358	0.661	0.292

Note: Due to the large number of data results, only the results of some years are listed; S1 represents the power generation efficiency of the first link; S2 represents the power transmission and distribution network efficiency of the second link.

**Table 4:** Scope of China’s six regional power grids.

Region	Regional Range	Region	Regional Range
North China	Beijing, Tianjin, Hebei, Shanxi, Shandong	Central China	Jiangxi, Henan, Hubei, Hunan, Chongqing, Sichuan
Northeast	Inner Mongolia, Liaoning, Jilin, Heilongjiang	South China	Guangdong, Guangxi, Hainan, Guizhou, Yunnan
Eastern China	Shanghai, Jiangsu, Zhejiang, Anhui, Fujian	Northwest	Shaanxi, Gansu, Qinghai, Xinjiang

**Table 5:** The improvement of input-output indicators in 30 provinces and cities in China in 2019.

Province	X_1_	X_2_	X_3_	X_4_	X_5_	Y_1_	Y_2_	Y_3_	U_1_	U_2_	U_3_	I_0_
Beijing	0.0	0.0	0.0	0.0	0.0	0.0	0.0	0.0	0.0	0.0	0.0	0.0
Tianjin	−1.0	−222.4	−83.5	0.0	0.0	0.0	0.0	333.5	0.0	−21.0	−4.7	−7.7
Hebei	−8.4	−5997.0	−1147.4	0.0	−31,578.3	0.0	0.0	946.5	−0.1	−47.7	−64.1	121.6
Shanxi	−12.3	−5434.3	−569.7	−5254.6	−65,166.7	0.0	0.0	0.0	−0.5	−172.2	−7.3	−1901.5
Inner Mongolia	−10.1	−9969.1	−883.8	−7099.7	−92,299.3	0.0	3856.9	0.0	−0.6	−284.5	0.0	−3315.7
Liaoning	−10.1	−2037.9	−193.7	−960.8	−32,781.7	0.0	0.0	0.0	−0.1	−57.2	0.0	−399.9
Jilin	−6.6	0.0	−32.5	−366.5	−24,682.5	698.9	0.0	0.0	−0.1	−24.7	0.0	−0.3
Heilongjiang	−7.3	−1761.3	−79.5	0.0	−27,356.5	575.1	0.0	300.7	−0.1	−17.6	0.0	244.0
Shanghai	0.0	0.0	0.0	0.0	0.0	0.0	0.0	0.0	0.0	0.0	0.0	0.0
Jiangsu	0.0	0.0	0.0	0.0	0.0	0.0	0.0	0.0	0.0	0.0	0.0	0.0
Zhejiang	−1.5	−3667.9	−105.2	−202.9	0.0	0.0	2887.5	0.0	0.0	−29.7	0.0	−357.9
Anhui	−3.5	−4630.6	−276.1	0.0	−29,158.2	0.0	0.0	1455.1	−0.3	−96.2	0.0	−425.4
Fujian	−4.2	−3289.0	−273.7	−249.1	−505.0	0.0	0.0	0.0	−0.1	−55.9	0.0	−578.7
Jiangxi	−4.3	−2539.4	−106.3	0.0	−20,275.0	0.0	0.0	426.4	−0.1	−28.6	0.0	−126.4
Shandong	−14.3	−4738.7	−778.9	−1520.6	−14,717.6	1089.5	0.0	1497.6	−0.2	−151.8	−3.6	−532.4
Henan	−12.4	−5839.1	−1036.4	0.0	0.0	0.0	0.0	1729.1	−0.1	−48.0	−52.7	520.2
Hubei	−6.7	−3447.9	−197.6	−1823.1	−16,670.7	0.0	0.0	0.0	−0.1	−45.4	0.0	−793.8
Hunan	−9.2	−1923.1	−438.7	−97.8	−30,484.9	0.0	0.0	0.0	0.0	0.0	0.0	0.0
Guangdong	0.0	0.0	0.0	0.0	0.0	0.0	0.0	0.0	0.0	0.0	0.0	0.0
Guangxi	−4.1	−2155.0	−285.0	−340.9	−37,028.2	0.0	0.0	0.0	−0.1	−33.2	0.0	−267.7
Hainan	0.0	0.0	0.0	0.0	0.0	0.0	0.0	0.0	0.0	0.0	0.0	0.0
Chongqing	−2.3	−974.1	−0.4	0.0	−13,270.2	214.7	0.0	0.0	0.0	−5.6	0.0	80.8
Sichuan	0.0	0.0	0.0	0.0	0.0	0.0	0.0	0.0	0.0	0.0	0.0	0.0
Guizhou	−6.0	−416.7	−121.5	−1628.7	−40,810.3	0.0	0.0	0.0	−0.4	−87.6	0.0	−728.8
Yunnan	0.0	0.0	0.0	0.0	0.0	0.0	0.0	0.0	0.0	0.0	0.0	0.0
Shaanxi	−10.0	−3202.2	−549.4	−3032.4	−51,157.6	0.0	0.0	0.0	−0.3	−106.1	0.0	−1167.5
Gansu	−6.6	−1496.8	0.0	−1928.5	−39,569.5	120.7	0.0	0.0	−0.1	−43.6	0.0	−482.8
Qinghai	0.0	0.0	0.0	0.0	0.0	0.0	0.0	0.0	0.0	0.0	0.0	0.0
Ningxia	−0.4	−2357.0	−68.6	−3163.6	−8627.9	71.0	1980.8	0.0	−0.2	−99.4	0.0	−912.1
Xinjiang	−6.7	−4935.3	−418.2	−5919.3	−71,228.0	0.0	0.0	0.0	−0.5	−231.3	−37.4	−2236.2

Note: X_1_, the number of employees in the power industry (10,000 people); X_2_, coal consumption for power generation (10,000 tons of standard coal); X_3_, fixed asset investment (100 million yuan); X_4_, the installed capacity of power generation (ten thousand Kw); X_5_, the length of the transmission lines (Km); Y_1_, the amount of electricity sold (100 million Kwh); Y_2_, the number of users served (households); Y_3_, clean energy power supply (100 million Kwh); U_1_, pollutant index; U_2_ represents plant power consumption (100 million Kwh); U_3_, line loss (100 million Kwh); I_0_, online power (100 million Kwh).

## Data Availability

The study did not report any data.
